# Climate Change Influences on the Global Potential Distribution of the Mosquito *Culex quinquefasciatus*, Vector of West Nile Virus and Lymphatic Filariasis

**DOI:** 10.1371/journal.pone.0163863

**Published:** 2016-10-03

**Authors:** Abdallah M. Samy, Arwa H. Elaagip, Mohamed A. Kenawy, Constância F. J. Ayres, A. Townsend Peterson, Doaa E. Soliman

**Affiliations:** 1 Entomology Department, Faculty of Science, Ain Shams University, Abbassia, Cairo 11566, Egypt; 2 Biodiversity Institute, University of Kansas, Lawrence, KS 66045, United States of America; 3 Department of Parasitology and Medical Entomology, Faculty of Medical Laboratory Sciences, University of Khartoum, Khartoum, Sudan; 4 Entomology Department, Centro de Pesquisas Aggeu Magalhães, Fundação Oswaldo Cruz, Recife-PE, Brazil; Cary Institute of Ecosystem Studies, UNITED STATES

## Abstract

Rapid emergence of most vector-borne diseases (VBDs) may be associated with range expansion of vector populations. *Culex quinquefasciatus* Say 1823 is a potential vector of West Nile virus, Saint Louis encephalitis virus, and lymphatic filariasis. We estimated the potential distribution of *Cx*. *quinquefasciatus* under both current and future climate conditions. The present potential distribution of *Cx*. *quinquefasciatus* showed high suitability across low-latitude parts of the world, reflecting the current distribution of the species. Suitable conditions were identified also in narrow zones of North Africa and Western Europe. Model transfers to future conditions showed a potential distribution similar to that under present-day conditions, although with higher suitability in southern Australia. Highest stability with changing climate was between 30°S and 30°N. The areas present high agreement among diverse climate models as regards distributional potential in the future, but differed in anticipating potential for distribution in North and Central Africa, southern Asia, central USA, and southeastern Europe. Highest disparity in model predictions across representative concentration pathways (RCPs) was in Saudi Arabia and Europe. The model predictions allow anticipation of changing distributional potential of the species in coming decades.

## Introduction

Mosquitoes are well known as vectors of many human and animal pathogens worldwide. The world has seen recent outbreaks and emergences of several tropical diseases caused by arboviruses and transmitted by mosquitoes. Species of the *Culex pipiens* complex transmit major etiological agents of human and animal diseases: West Nile virus (WNV), Saint Louis encephalitis virus (SLEV), Sindbis virus, Rift Valley fever virus (RVFV) and lymphatic filariasis (LF) [1, 2]. *Cx*. *pipiens* is the most widely distributed mosquito worldwide. It comprises a complex of subspecies or forms, including *Cx*. *pipiens* Linnaeus 1758, *Cx*. *quinquefasciatus* Say 1823, *Cx*. *pipiens pallens* Coquillett 1898, and *Cx*. *australicus* Dobrotworsky & Drummond 1953. These mosquitoes are closely associated with human disease in many regions [3, 4]. The *Cx*. *pipiens* complex is viewed as a questionable point in mosquito taxonomy, because species (or forms) are indistinguishable morphologically and can be separated only by molecular analysis [5, 6] or details of behavioral and physiological characteristics [7].

In terms of geographic distribution, *Cx*. *quinquefasciatus* differs from *Cx*. *pipiens* in that the former is most prevalent in tropical and sub-tropical areas [8, 9]. In the southern United States, *Cx*. *quinquefasciatus* is the primary vector of Saint Louis encephalitis virus and West Nile virus [10–12]. *Culex quinquefasciatus* is identified as the major vector of the filarial nematode, *Wuchereria bancrofti* (Cobbold, 1877) in Brazil [13], tropical Africa, and Southeast Asia [12], and RVFV in Africa [14, 15].

Recently, Ayres [16] raised the possibility of *Cx*. *quinquefasciatus* may be involved in Zika virus (ZIKV) urban transmission in Brazil, where its abundance is approximately 20-fold higher than the known ZIKV vector, *Aedes aegypti*. ZIKV infection has been associated with neurological complications, such as Guillain-Barré syndrome and also with a severe malformation, fetal microcephaly [17, 18]. Currently, ZIKV is spreading globally, and ZIKV outbreaks have been reported in 65 countries [19]. Experimental studies of vector competence have confirmed that *C*. *quinquefasciatus* can disseminate and transmit ZIKV [20]. Ongoing projects are attempting to detect ZIKV in natural *Culex* populations in areas where epidemics are occurring, to provide the final piece of evidence for this hypothesis.

Vector-borne diseases are vulnerable to climate changes and may emerge in response to global warming [21], such that patterns of transmission of WNV and other diseases are likely to change in coming decades [22]. This effect may result from expansions of vector ranges, which place non-endemic areas at risk if sources of infections are available [23]. Studies of field populations of *Culex* mosquitoes in general have revealed that increases in temperature are likely to accelerate mosquito development [24], increase vector abundance, and lead to emergence of diseases [22]. For example, in WNV epidemiology [25] rising temperature and changes in rainfall allowed circulation of WNV in different areas in southern USA, Europe, western Asia, and the eastern Mediterranean [26]. In addition, transmission of WNV can be accelerated with increasing temperatures, as demonstrated by Kilpatrick et al. (2008) [27] for West Nile Virus in *Culex pipiens*.

Important knowledge gaps remain regarding effects of climate and climate change on emergence of several vector-borne diseases in the world. Here, we provide detailed global maps of current potential distributions of *Cx*. *quinquefasciatus*, the potential vector for WNV, SLEV, and LF, and examine possible changes in the potential distribution of the species under future climatic conditions, based on outputs of 11 general circulation models (GCMs) and 4 representative concentration pathways (RCPs).

## Materials and Methods

### Occurrence data

Occurrence records for *Culex quinquefasciatus* were obtained from VectorMap (www.vectormap.org), the Global Biodiversity Information Facility (GBIF;www.gbif.org), and the PubMed database using the search term “*Culex quinquefasciatus*”. We included all records with geographic coordinates, and filtered data to eliminate duplicate records in the final data set. The final records of *Cx*. *quinquefasciatus* were divided into two halves: 50% for calibrating ecological niche models for the species, and 50% for evaluating predictions of those models.

### Climatic data

Data from WorldClim (www.worldclim.org) were used to characterize current global climates, including 19 bioclimatic variables originally derived from monthly temperature and rainfall values collected from weather stations in 1950–2000 [28]. The data are available at three spatial resolutions; we selected the coarsest (10’), in light of the global extent of our model calibration area. To characterize influences of climate change on the distribution of *Cx*. *quinquefasciatus*, we selected parallel data sets for four representative concentration pathways (RCPs; RCP 2.6, RCP 4.5, RCP 6.0, and RCP 8.5) accounting for different future emission scenarios from the Coupled Model Intercomparison Project Phase 5 (CMIP5) available in WorldClim archive. For each RCP, we included 11 GCMs for which data for all RCPs were available, for a total of 44 combinations (S1 File). Bioclimatic variables 8–9 and 18–19 were omitted from analysis, in light of known spatial artifacts in those variables. The remaining of 15 variables were submitted to a principle components analysis (PCA) to reduce the dimensionality and avoid multicollinearity between variables [29]. The component loadings in the present-day data were used to transform future-climate data using the PCAProjection function in ENMGadgets [30] in R software version 3.2.0 [31].

### Ecological niche modeling

The Grinnellian ecological niche of *Cx*. *quinquefasciatus* was estimated using the maximum entropy algorithm implemented in Maxent v3.3.3e [32]. The Grinnellian niche is characterized as the set of environmental conditions needed by the species to maintain populations without immigrational subsidy [29]. The models were calibrated based on the first six principal components from the PCA analysis described above, and then transferred to our 44 views of potential future conditions. We ran 100 bootstrap replicates in Maxent, and the median output was used in analyses. The median of medians across all GCMs for each RCP was as a best guess of conditions under that RCP, and final models were thresholded based on a maximum allowable omission error rate of 5% (*E* = 5%; [33]), assuming that up to 5% of occurrence data may include errors that misrepresented environmental values. Uncertainty associated with the models was estimated as the range (maximum–minimum) of suitability across models for each RCP [34].

The model performance was evaluated using partial receiver operating characteristic (ROC) statistics applied to the 50% subset of occurrences left out before model calibration for testing. This approach avoids possible errors raised with traditional ROC provided in Maxent outputs [35]. Partial ROC statistics was calculated using the PartialROC function available in ENMGadgets package.

## Results

**Fig 1:**
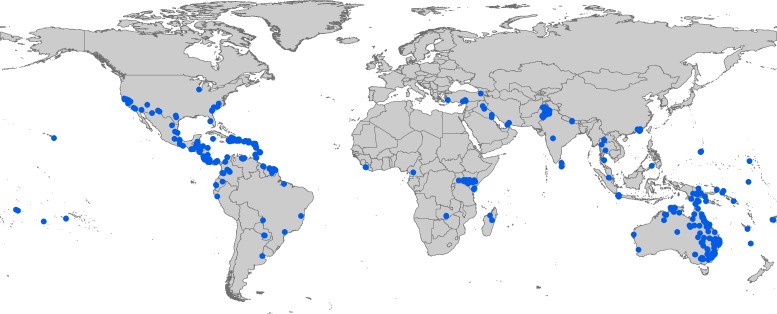
Summary of 701 *Culex quinquefasciatus* occurrences available for model calibration.

We assembled 1402 occurrence records for *Cx*. *quinquefasciatus*. The full data set is available at https://dx.doi.org/10.6084/m9.figshare.3487046. Overall, the distribution was concentrated on southern continents, although the species was well represented in North America and southern Asia (Fig 1).

**Fig 2:**
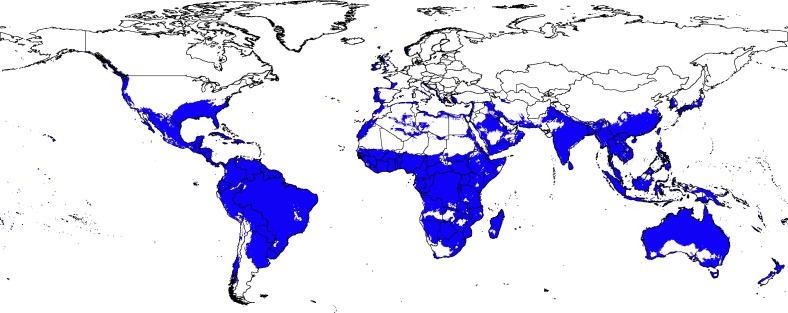
Current potential distribution of *Culex quinquefasciatus* based on present-day climatic conditions. Blue shaded areas were modeled as suitable; white areas were modeled as unsuitable.

The potential distribution of *Cx*. *quinquefasciatus* under present-day conditions showed high suitability across southern North America, much of South America, sub-Saharan Africa, south Asia, and most of Australia and New Zealand (Fig 2). Parts of West Africa, Western Europe, and East Asia were modeled as suitable environmentally, although few occurrence points came from these areas. Model predictions performed better than random expectations, based on the partial ROC test (*P* < 0.001).

**Fig 3:**
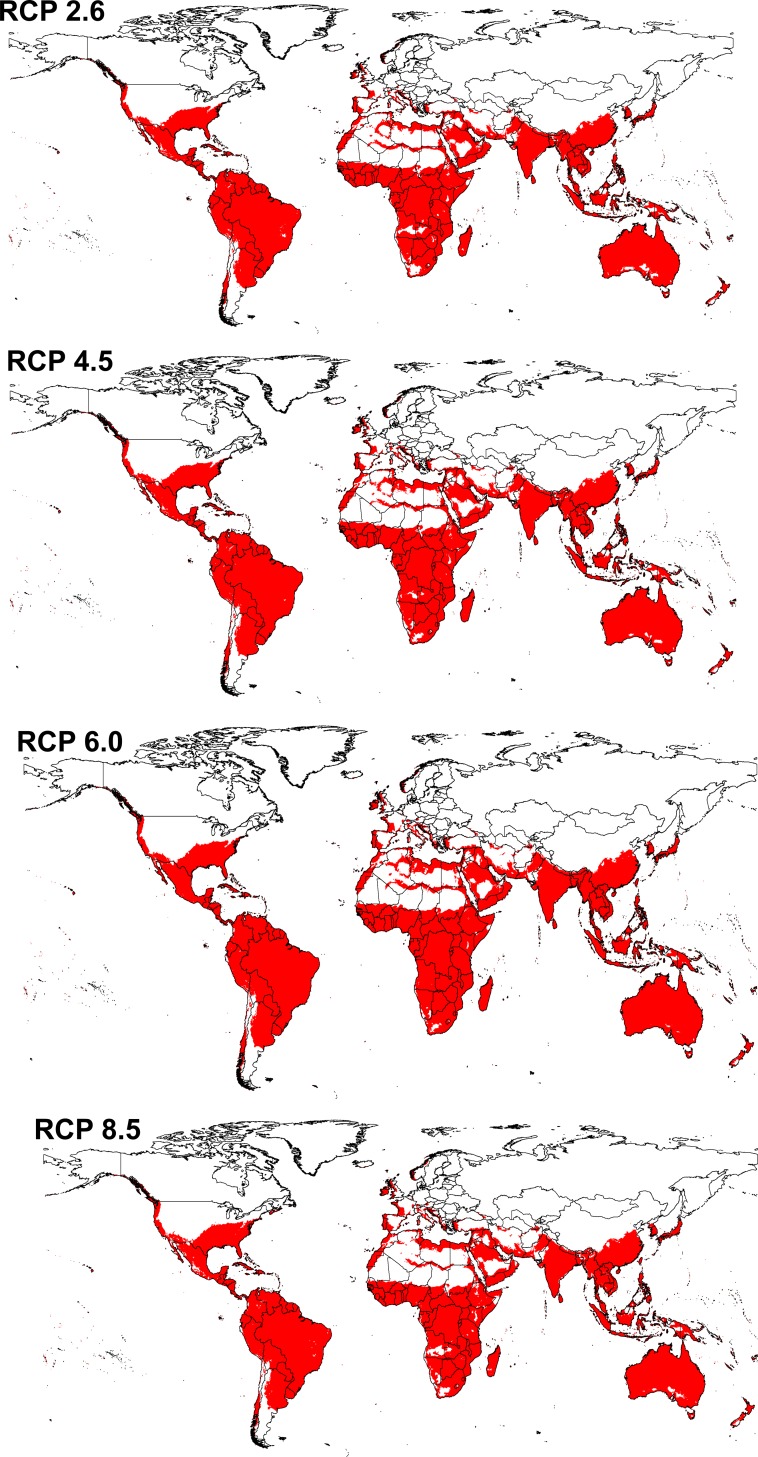
Predicted future potential distribution of *Culex quinquefasciatus* under four future representative concentration pathways of climate conditions. Red areas are modeled suitable conditions; white areas are unsuitable conditions.

Transferring the *Cx*. *quinquefasciatus* model to future conditions showed an overall distributional pattern similar to that under present-day conditions; however, the species showed higher suitability in southern Australia under future conditions (Fig 3). The future potential distribution was thus estimated as including the southern United States, Central and South America, central and southern Africa, South Asia, and Australia.

**Fig 4:**
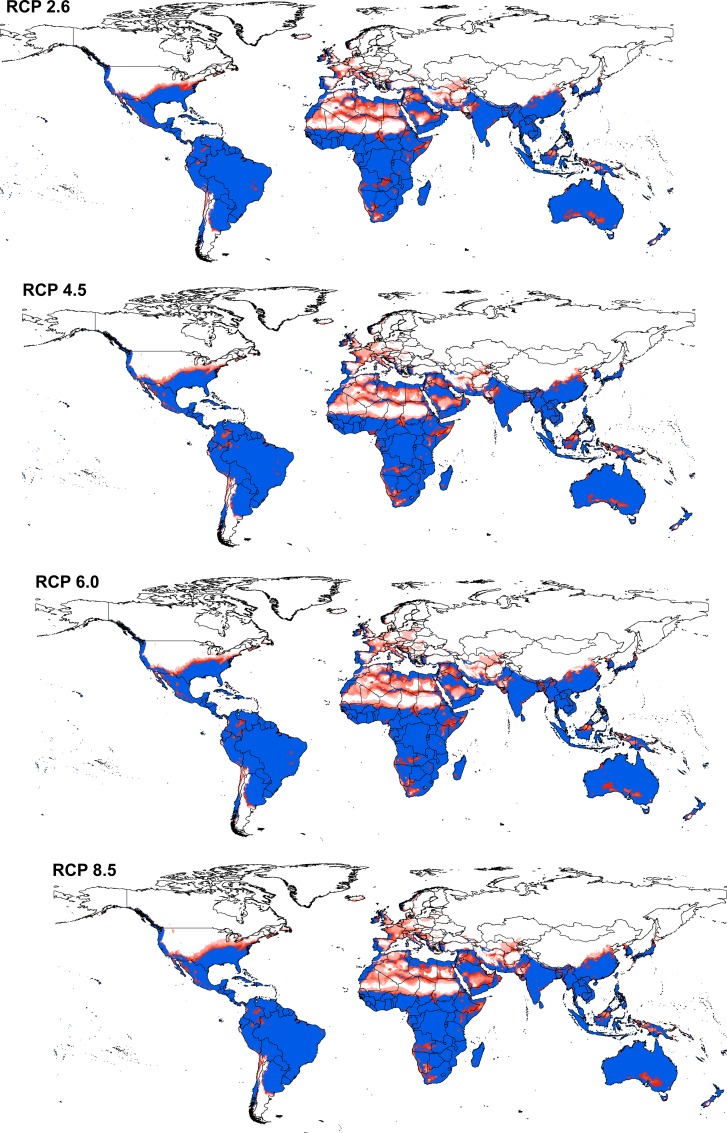
Summary of the modeled global distribution of *Culex quinquefasciatus* under both current and future climatic conditions to show the stability of predictions at present and into the future, and to illustrate differences among representative concentration pathways (RCPs). Dark blue represents model stability under both current and future conditions, dark red represents agreement among all climate models in anticipating potential distributional areas in the future, and light red indicates low agreement between diverse climate models as regards distributional potential in the future.

The potential distributional area of *Cx*. *quinquefasciatus* increased from present-day conditions to RCP 6.0, and then decreased in RCP 8.5. Area increased by 4.9% from present-day conditions to RCP 6.0 then decreased by 1.3% from RCP 6.0 to RCP 8.5 (details for each GCM are presented in supporting information 2). Detailed maps of *Cx*. *quinquefasciatus* model stability in coming decades (Fig 4) illustrate differences among RCPs. Highest stability of the models among present-day and future conditions appeared in the belt between 30°S and 30°N, which includes much of South America, central and southern Africa, South and East Asia, Australia, and New Zealand. The same pattern of suitability was also observed in a narrow zone in Western Europe and the southern United States. Areas presenting full agreement among all future climate models in anticipating distributional potential in the future include only Kangaroo Island (Australia), Somalia, and Colombia. Areas showing low agreement (= high uncertainty) among climate models as regards distributional potential in the future included North and Central Africa, Afghanistan, Pakistan, the central United States, and southeastern Europe. Interactive maps for present-day and future distribution of *Cx*. *quinquefasciatus* are presented in the supplementary materials (S3–S7 Files).

**Fig 5:**
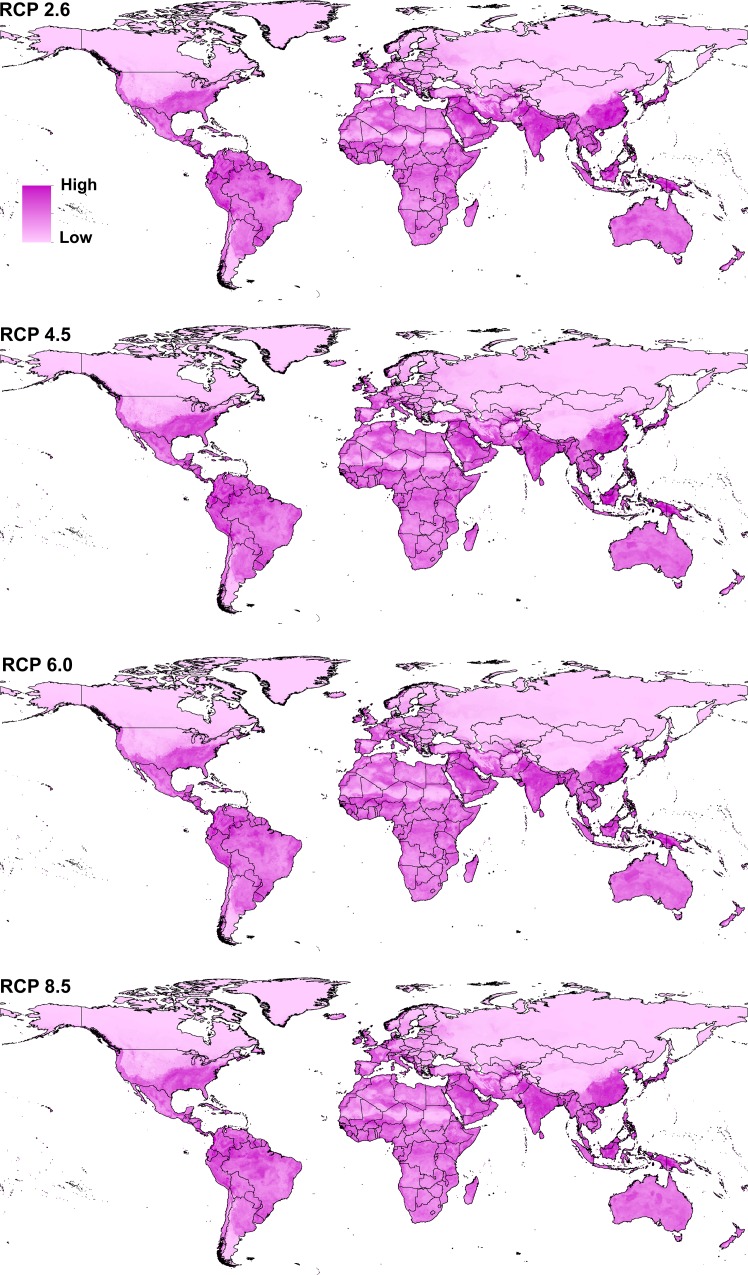
Uncertainty estimates associated with *Culex quinquefasciatus* niches models under future conditions represented by four representative concentration pathways.

The study provided uncertainty estimates associated with different circulation models in each RCP (Fig 5). Highest variation in model predictions across all RCPs was observed in East Asia, the Arabian Peninsula, central North America, western South America, and Europe.

## Discussion

This study assembled a global data set summarizing occurrences of *Cx*. *quinquefasciatus*, and provided detailed maps of its potential geographic distribution under current and future climatic conditions. The latter objective is important to anticipating any possible future distributional changes of *Cx*. *quinquefasciatus*. These maps (1) give a detailed picture of the current distribution of *Cx*. *quinquefasciatus*, which is a potential vector for several arboviruses and lymphatic filariasis; (2) anticipate possible changes in the range of the species under future conditions; (3) identify areas of risk where diseases transmitted by this vector can be established with availability of infection sources through human dynamics; and (4) identify countries with priorities for *Cx*. *quinquefasciatus* surveillance programs where data are unavailable (e.g., Western Europe). The predicted distribution of the species was focused in southern regions of the world, both under present-day and future conditions.

However, closely related species of *Culex* mosquitoes are distributed differently [36], and hybrid zones of *Cx*. *pipiens* and *Cx*. *quinquefasciatus* have been reported to occur in Madagascar, North America, and Argentina [5, 37, 38]. Hybrid zone areas in the eastern United States [39] were identified as showing high suitability for occurrence of *Cx*. *quinquefasciatus* in our study.

No previous reports have placed the species in Europe or North Africa; the closest area where the species occurred was in Turkey [40]. Our models revealed environmental suitability for the species’ occurrence in parts of Europe and North Africa. Hence, either the species is present there but not documented owing to difficulties in morphological identification of the members of species complex (i.e. the species in the complex are nearly morphologically identical; [5]), or it is absent but vulnerable to possible introduction from Turkey or elsewhere. The behavior and physiology of *Cx*. *pipiens* complex in Europe and USA are different [41], and gene flow between species in the complex has been reported [41].

The global distribution of *Cx*. *quinquefasciatus* presents a risk for introduction and transmission of WNV in novel areas [41], such as Brazil, Peru, Australia, and New Zealand. Possible expansion of the range of *Cx*. *quinquefasciatus* may place still more countries at risk of exposure: for example, higher summer temperatures have been identified as a key factor associated with WNV expansion in British Columbia in Canada [42]. WNV outbreaks in Europe have been nonrecurring and localized; however, they have been enzootic and widespread in USA [41]. This pattern of disease spread may be a reflection to the distributional pattern of key vector populations or may reflect the recency of its establishment in North America.

Early studies suggested that warmer conditions are drivers of mosquito abundance [43–45]; however, other studies suggested a delay in the start of the breeding season of *Cx*. *quinquefasciatus* in sites presenting a dry and hot spring and summer, but extensions in the season with fall rains and higher temperatures [46]. Our prediction suggested suitability of occurrence of *Cx*. *quinquefasciatus* in regions with lower temperatures than in tropical and subtropical regions. Generally, *Cx*. *quinquefasciatus* is likely to experience decreased survival as a result of elevated temperatures [47]. A previous study showed a tripling in rates of development, fecundity, and feeding with higher temperature [47]. Although climate change likely will affect the biology of *Cx*. *quinquefasciatus* directly, distributional changes in response to elevated temperatures will likely be manifested. Climate change can thus trigger changes in the distributional patterns of *Cx*. *quinquefasciatus*, but these changes will be strongly dependent on the location and timing of climate changes [46]. Other environmental effects are on survival, reproductive rate, and vectorial capacity to transmit pathogens; for example, higher temperatures increase pathogen proliferation, and therefore vector competence [27, 48, 49].

Understanding vector distributions is important to understanding dynamics of pathogen transmission. In our analyses, suitable areas were identified for *Cx*. *quinquefasciatus* in central and southern Africa, Madagascar, and North Africa (i.e., northern Egypt, Libya, Tunisia, and Morocco). North African countries should be considered as priority in surveillance of this species. Establishment of integrated surveillance programs to improve mosquito data was one of the objectives for the MediLabSecure project launched for the Balkan region [50].

## Conclusions

The integrated maps of current and future distributions of *Cx*. *quinquefasciatus* can guide better applications of vector surveillance and disease control programs across the world. The distributional maps of *Cx*. *quinquefasciatus* can be also useful to the Global Lymphatic Filariasis Elimination Programme (GLFEP), which considers control of *Cx*. *quinquefasciatus* as an important element in control efforts in most endemic countries [51]. These maps are also key elements in recent events in which arboviruses have emerged worldwide. This study can guide control programs and surveillance priorities, which are primarily dependent on identification of suitable areas where the vector occurs or may occur. Finally, it is important to highlight that if *Cx*. *quinquefasciatus* is proven to be functioning as a ZIKV vector, the disease control strategies will change dramatically, since most affected countries have no control program targeting this species. In this case, these maps will form a baseline by which to anticipate areas at ZIKV risk and will help in response to the disease outbreak.

## Supporting Information

S1 File A summary of general circulation models used for estimating the potential distribution of *Culex quinquefasciatus* based on future climatic conditions. (CSV)

S2 File Expansion or contraction of *Culex quinquefasciatus* ranges based on presence-absence matrix from the estimated ecological niche for each climate model in four representative concentration pathways. (CSV)

S3 File Interactive map for the current potential distribution of *Cx*. *quinquefasciatus*. (KMZ)

S4 File Interactive map for the predicted distribution of *Cx*. *quinquefasciatus* in representative concentration pathway 2.6. (KMZ)

S5 File Interactive map for the predicted distribution of *Cx*. *quinquefasciatus* in representative concentration pathway 4.5. (KMZ)

S6 File Interactive map for the predicted distribution of *Cx*. *quinquefasciatus* in representative concentration pathway 6.0. (KMZ)

S7 File Interactive map for the predicted distribution of *Cx*. *quinquefasciatus* in representative concentration pathway 8.5. (KMZ)
